# Association between oxidative balance score and metabolic syndrome and its components in US adults: a cross-sectional study from NHANES 2011–2018

**DOI:** 10.3389/fnut.2024.1375060

**Published:** 2024-03-13

**Authors:** Yi Lu, Meixiang Wang, Jiaxin Bao, Dashuang Chen, Hao Jiang

**Affiliations:** ^1^Department of Cardiology, The Affiliated Taizhou People’s Hospital of Nanjing Medical University, Taizhou School of Clinical Medicine, Nanjing Medical University, Taizhou, China; ^2^Department of Nephrology, The Affiliated Taizhou People’s Hospital of Nanjing Medical University, Taizhou School of Clinical Medicine, Nanjing Medical University, Taizhou, China; ^3^Department of General Medicine, The Affiliated Taizhou People’s Hospital of Nanjing Medical University, Taizhou School of Clinical Medicine, Nanjing Medical University, Taizhou, China

**Keywords:** oxidative balance score (OBS), metabolic syndrome, NHANES, oxidative stress, antioxidant

## Abstract

**Background:**

Metabolic syndrome (MetS) is a global health concern that threatens human well-being. The oxidative balance score (OBS) is a tool to identify the level of oxidative stress that is correlated with MetS risk. However, association between OBS and MetS and its components has not been reported.

**Methods:**

This cross-sectional study included adult individuals with complete OBS and MetS data from National Health and Nutrition Examination Survey (NHANES) 2011–2018. A weighted logistic regression analysis was conducted to identify the association of the total, dietary, and lifestyle OBS scores with MetS. Subgroup analyses and restricted cubic splines were used to further explore associations.

**Results:**

In total, 10,025 eligible adult individuals (51.48% were males at a median age of 46 years) were included, with a MetS prevalence of 29.98%. In fully adjusted model, higher total OBS was associated with reduced risk of MetS (Q3 vs. Q1: odds ratio [OR] = 0.57, 95% confidence interval [CI], 0.46–0.71, *p* < 0.001; Q4 vs. Q1: OR = 0.42, 95% CI, 0.33–0.53, *p* < 0.001; P for trend <0.001). Significant differences were observed in the relationship between dietary OBS and lifestyle OBS and MetS risk (continuous dietary OBS: OR = 0.97, 95% CI, 0.96–0.98, *p* < 0.001; continuous lifestyle OBS: OR = 0.61, 95% CI, 0.58–0.64, p < 0.001), as well as negative relationship between total OBS and risk of five MetS components (all *p* < 0.05). Subgroup analysis showed marital status modified the negative association between OBS and MetS in subgroup analysis (P for interaction = 0.014). Moreover, a nonlinear correlation between OBS and MetS (including its components) was found, further elucidating associations (all *p* < 0.05). Restricted cubic splines demonstrated not obviously U-shape correlation between OBS and MetS components (elevated triglyceride and blood pressure).

**Conclusion:**

This study suggests a strong association between the OBS and MetS and its components. Our data indicated that a higher OBS score was correlated with a decreased risk of MetS and its components in a nonlinear manner. Hence, the OBS may serve as an effective marker for identifying individuals with MetS, with a higher score indicating a predominance of more antioxidants.

## 1 Introduction

Metabolic syndrome (MetS) is defined by a constellation of interrelated factors, with hyperlipidemia, elevated blood pressure (BP), and dysregulated serum glucose, serving as its primary components. Central obesity and insulin resistance (IR) play vital roles in the manifestations of MetS (1). The prevalence of MetS has significantly surged in recent years owing to various factors, including lifestyle-related elements and unhealthy dietary patterns, which pose a threat to human well-being and quality of life worldwide (2). According to current statistics, approximately 35% of adults in the United States (US) have MetS (3).

Previous studies have shown that oxidative stress may play a role in the onset and progression of MetS (4–6). Oxidative stress may occur when there is an imbalance between pro-oxidants and antioxidants, leading to damage to macromolecules and disruption of redox signaling and control (7). Exogenous modifiable factors, such as diet, lifestyle, and medications, play a key role in the oxidative balance in the body (8). Factors augmenting the levels of reactive oxygen species (ROS) through diverse mechanisms are considered prooxidants, including smoking, alcohol, iron, lipid-rich diets, and ionizing radiation (9–13). Antioxidants are proved as efficacious interventions for mitigating oxidative stress (14). By governing the generation of ROS and reactive nitrogen species and regulating signal transduction pathways (15), various dietary nutrients exhibit antioxidant properties, such as carotenoids, the Vitamin B family, Vitamin C, Vitamin E, and certain metals (11, 16–18). In addition to diet, physical activity can alleviate oxidative stress by enhancing the antioxidant response through the activation of Nrf2, reducing systemic oxidative stress markers and promoting an increase in antioxidant enzymes and nitric oxide (NO) availability (19).

The complex interplay and associations between prooxidant and antioxidant factors present a formidable obstacle to discerning their individual contributions to the risk of disease. It has been observed that the combined presence of anti/pro-oxidant constituents exerts a more pronounced influence on metabolic disorders than any singular anti/pro-oxidant factor, primarily by modulating the levels of oxidative stress within the body (2). To comprehensively assess the equilibrium of an individual’s oxidation–reduction status, Goodman et al. introduced the oxidative balance score (OBS), a scoring system that quantifies oxidative stress levels and provides a semi-quantitative measure of the oxidative balance in the body (20). Subsequently, an increasing number of OBSs that employ diverse scoring criteria have been proposed to assess the effects of oxidative status on various diseases (21). In our study, the OBS incorporated 16 dietary and 4 lifestyle factors. Assigning values of 0, 1, and 2 for high-to-low pro-oxidant exposure, and 2, 1, and 0 for high-to-low antioxidant exposure, we summed these individual scores to calculate the overall OBS. Higher and lower scores indicated shifts towards antioxidant and pro-oxidant exposure, respectively (22). We selected this scoring system for its comprehensive evaluation of diet and lifestyle impacts and semi-quantitative assessment of various oxidants and antioxidants, providing a more holistic and direct observation of exogenous antioxidant effects on the body’s oxidative balance.

Based on previous studies, lower scores across all OBSs indicate higher oxidative stress, which is associated with adverse clinical outcomes (21). The relationships between OBS and hypertension (23), diabetes (24), nonalcoholic fatty liver disease (25), cancer (26), all-cause mortality (27) and biological aging (28) have been intensively investigated and widely discussed. However, relatively few studies probe the correlation between attitudes toward OBS and MetS risk. A large-scale cross-sectional study conducted in South Korea demonstrated that the OBS was inversely correlated with MetS stratified by sex (29). An Iranian study concluded no significant association of OBS with MetS (2). To date, no study has evaluated the correlation between the OBS (including dietary and lifestyle OBS) and MetS or its components. Therefore, the study aimed to investigate the potential relationship between OBS and MetS and its components in the US population to provide insights into the prevention of MetS in individuals through OBS.

## 2 Materials and methods

### 2.1 Study design and population

The National Health and Nutrition Examination Survey (NHANES) is a nationwide survey with multistage sampling design that aims to investigate the nutritional and health status of adults and children in the US. The survey protocol was authorized by the National Center for Health Statistics, and written consent was obtained (30). Details of NHANES are provided on its official website.

The study was performed based on four consecutive survey cycles from NHANES 2011–2018. Of the initial 39,156 participants enrolled, those younger than 18 years (*n* = 15,331) were excluded. Participants with missing dietary and lifestyle OBS data (*n* = 11,988) and those with missing MetS data (*n* = 5,419) were excluded. Individuals with missing covariate data (*n* = 1,547) were excluded. Finally, 10,025 eligible participants were included in the study.

### 2.2 Exposure definition

We constructed and calculated OBS on the basis of previous studies. In this study, the OBS was composed of four lifestyle factors and 16 dietary components (13, 21). The dietary factors were classified into prooxidants (total fat and iron) and antioxidants (β-carotene, dietary fiber, copper, vitamin B6, vitamin B12, vitamin C, niacin, vitamin E, total folate, vitamin B2, magnesium, calcium, zinc, and selenium) according to the effect on oxidative stress. Lifestyle factors were classified as prooxidants (alcohol intake, body mass index, and cotinine) and antioxidants (physical activity). Dietary OBS components were assessed in the NHANES using 24-h food recalls. Physical activity, expressed as weekly metabolic equivalents (MET), was calculated using data on leisure time activities over the past 30 days acquired from household interviews (22).

The detailed criteria for the Oxidative Balance Score (OBS) were delineated in Supplementary Table S1. Prooxidant dietary and lifestyle factors were stratified into tertiles and assigned scores ranging from 0 to 2, corresponding to the lowest to highest tertile, respectively. Conversely, antioxidant dietary and lifestyle factors were also categorized into tertiles, and scores were inversely assigned from 2 to 0, reflecting the highest to lowest tertile of antioxidants. The total OBS was derived by aggregating scores across all individual components, encompassing both dietary and lifestyle components. Specifically, the dietary OBS was computed based on the allocation of dietary prooxidants and antioxidants, whereas the lifestyle OBS was determined through the assessment of lifestyle-related prooxidants and antioxidants. Notably, participants with alcohol intake were divided into heavy drinkers [(≥ 30 g/d for male and ≥ 15 g/d for female), nonheavy drinkers (0–30 g/d for male and 0–15 g/d for female)] and nondrinkers, who were given 0, 1, and 2 score, respectively. Physical activity was assigned a score of 0 for <400 METs-min/week, 1 score for 400–1,000 MET-min/week, and 2 for >1,000 METs-min/week.

### 2.3 Outcome definition

MetS was characterized based on the criteria proposed by Adult Treatment Program III of the National Cholesterol Education Program (31). Participants were considered to be diagnosed with MetS when three or more than three following criteria were met: (1) Elevated triglyceride (TG): serum TG ≥150 mg/dL (1.69 mmol/L) or specific drug treatment; (2) Low high density cholesterols (HDL-C): serum HDL-C) < 40 mg/dL (1.03 mmol/L) in men and < 50 mg/dL (1.29 mmol/L) in women or specific treatment; (3) Elevated fasting plasma glucose (FPG): PFG ≥ 110 mg/dL (6.1 mmol/L) or drug treatment of previously diagnosed type 2 diabetes; (4) Elevated waist circumference (WC): WC ≥ 102 cm in men or ≥ 88 cm in women; (5) Elevated BP: systolic BP ≥ 130 mmHg or diastolic BP ≥ 85 mmHg or drug treatment of previously diagnosed hypertension.

Blood samples were obtained following an overnight fast from the participants. A Cobas C Chemistry Analyzer (C311, Roche Co.) was used to measure plasma glucose levels. Additionally, serum concentrations of HDL-C and TG were measured, using Cobas C Chemistry Analyzer (6,000, Roche Co.). Body measurements, such as height, weight, and waist circumference (WC), were Energy intake and caffeine was extracted from the 24-h food recall Systolic and diastolic BP were both derived from the average of three repeated measurements after sitting for 5 min for all participants. Trained health technicians performed these measurements using a calibrated Omron IntelliSense Blood Pressure Monitor. Data of prescription medications and disease diagnosis was obtained using self-reported information from the NHANES questionnaire.

### 2.4 Covariates

Standardized questionnaires were administered to NHANES participants to obtain sociodemographic, dietary, and lifestyle information. We incorporated a series of covariates possibly associated with MetS to adjust for confounding effects, based on previous studies (32). The covariates included age continuous or categorical (18–39, 40–59, or ≥ 60 years), sex (male or female), race/ethnicity (non-Hispanic black, non-Hispanic white, Hispanic or other race), education levels (less high school, high school diploma or more than high school), marital status (married, divorced or living alone), family poverty-to-income ratio (PIR) as continuous or categorical variables (<1, 1–2, 2–4 or > 4), energy intake, caffeine, and sleep trouble (yes or no). Sleep trouble was measured by asking the participants, “Have you ever told a doctor or other health professional that you have trouble sleeping?” (SLQ050). Participants’ responses included “Yes,” “No,” “Refused,” and “Do not know.” Participants responding “yes” were defined with sleep trouble. “Refused” and “Do not know” were recorded as missing values. Energy intake and caffeine was extracted from the 24-h food recall.

### 2.5 Statistical analysis

All statistical analyses were conducted based on the Centers for Disease Control (CDC) recommendations. The sample weight in the study was the mean dietary day one 2-year sample weight (WTDRD2), which accounted for a complex sampling design. OBS was analyzed separately as total, lifestyle and dietary OBS. The same statistical analyses were performed on total, lifestyle and dietary OBS. The Anderson-Darling test was conducted to check the distribution of the continuous variables. Continuous variables with Gaussian distribution were presented as means with standard errors (SE). Continuous variables with non-Gaussian distribution were presented as medians with interquartile ranges (IQR). Categorical parameters were presented as proportions with SE. Differences between four groups were calculated using the Kruskal–Wallis H test (continuous variables with non-Gaussian distribution) and ANOVA tests (continuous variables with Gaussian distribution). Differences between groups were calculated using the chi-squared test for categorical variables. Univariate and multivariate weighted logistic regressions were conducted to identify the relationship between OBS (total, dietary, and lifestyle) and MetS (and its components). Model 1 was constructed without adjusted covariates. Model 2 was adjusted for age, sex, race, PIR, marital status, and educational level. Model 3 was built after adjusting for age, sex, race, PIR, marital status, education level, energy intake, caffeine intake, and sleep problems. The Spearman’s rank correlation coefficients were used to assess the correlation between dietary OBS and lifestyle OBS. A stratified analysis was then conducted to examine the association between the different subgroups of MetS. Interaction tests were conducted by adjusting all other covariates in different subgroups. Finally, a restricted cubic spline (RCS) analysis was performed to explore the nonlinear relationship between OBS and MetS (and its components). All statistical analyses were conducted in R 4.3.2 using “nhanesR,” “survey,” and “rms” package. Statistical significance was defined as a two-sided *p*-value <0.05.

## 3 Results

### 3.1 Baseline characteristics of the participants

A total of 10,025 individuals were included in the study, with males accounting for 51.48% of the population. The median age of the participants was 46 years, and 29.98% were diagnosed with MetS. Divided by quartiles with increasing OBS scores, the OBS was grouped into Q1, Q2, Q3, and Q4, with sample sizes of 2,702, 2,644, 2,417, and 2,262, respectively. The range of scores was <17 in the Q1 group, 17–22 in the Q2 group, 23–27 in the Q3 group, and > 27 in the Q4 group. As the quartiles gradually increased, a lower proportion of participants were diagnosed with MetS. Among the four quartiles of OBS, statistical significance was observed for race, education level, marital status, energy intake, and caffeine intake (all *p* < 0.001), but not for age, sex, and sleep trouble. Notably, the prevalence of MetS gradually decreased in the five components of MetS (elevated TG, low HDL-C, elevated FPG, elevated WC, and elevated BP) in the higher OBS quartiles. The baseline characteristics of these participants are shown in Table 1.

**Table 1:** Baseline characteristics of the participants.

Characteristics	Total	Q1	Q2	Q3	Q4	*p* value
Age (years)	47 (32, 59)	46 (31, 59)	48 (32, 60)	47 (33, 59)	46 (33, 58)	0.210
Gender, % (SE)						0.070
Male	51.48 (0.02)	53.93 (1.20)	52.98 (1.53)	49.27 (1.43)	49.86 (1.41)	
Female	48.52 (0.02)	46.07 (1.20)	47.02 (1.53)	50.73 (1.43)	50.14 (1.41)	
Race/Ethnicity, % (SE)						<0.001
Non-Hispanic Black	9.82 (0.01)	16.05 (1.46)	10.34 (1.06)	7.91 (0.80)	5.46 (0.62)	
Non-Hispanic White	68.86 (0.04)	64.63 (2.18)	64.63 (2.18)	70.75 (2.02)	71.83 (1.86)	
Hispanic	5.33 (0.01)	5.39 (0.68)	5.39 (0.68)	5.26 (0.62)	5.55 (1.86)	
Other race	15.99 (0.01)	13.93 (1.08)	13.93 (1.08)	16.08 (1.41)	17.16 (1.23)	
Education levels, % (SE)						<0.001
Less than high school	2.57 (0.01)	3.28 (0.35)	3.07 (0.38)	2.15 (0.31)	1.83 (0.31)	
High school diploma	7.22 (0.01)	11.16 (0.85)	7.83 (0.82)	5.50 (0.65)	4.68 (0.71)	
More than high school	90.20 (0.03)	85.55 (1.01)	89.10 (1.01)	92.35 (0.75)	93.49 (0.84)	
Marital status, % (SE)						<0.001
Married	55.18 (0.03)	46.64 (1.70)	35.18 (1.61)	32.90 (1.70)	28.84 (1.70)	
Divorced	10.46 (0.01)	12.33 (0.82)	10.74 (0.87)	10.05 (0.98)	8.85 (0.96)	
Living alone	34.36 (0.03)	41.04 (1.56)	35.18 (1.61)	57.05 (1.60)	62.30 (1.89)	
Poverty income ratio	3.3 (1.6–5.0)	2.4 (1.2–4.5)	3.1 (1.6–5.0)	3.4 (1.8–5.0)	4.0 (2.1–5.0)	<0.001
Energy intake (kcal)	2057 (1,537, 2,688)	1,500 (1,125, 1991)	1949 (1,510, 2,499)	2,217 (1713, 2,870)	2,517 (2018, 3,224)	<0.001
Caffeine (mg)	127 (33, 249)	105 (28, 228)	128 (35, 241)	144 (33, 260)	135 (29, 253)	<0.001
Sleep trouble, % (SE)						0.180
Yes	29.09 (0.01)	30.98 (1.49)	29.19 (1.55)	29.76 (1.54)	26.55 (1.35)	
No	70.92 (0.02)	69.02 (1.49)	70.81 (1.55)	70.24 (1.54)	73.45 (1.35)	
MetS, % (SE)						<0.001
Yes	29.98 (0.01)	35.51 (1.25)	34.71 (1.42)	27.46 (1.52)	22.54 (1.44)	
No	70.02 (0.03)	64.49 (1.25)	65.29 (1.42)	72.54 (1.52)	77.46 (1.44)	
Elevated TG, % (SE)						<0.001
Yes	36.31 (0.02)	38.80 (1.51)	39.78 (1.66)	34.64 (1.34)	32.11 (1.39)	
No	63.69 (0.02)	61.20 (1.51)	60.22 (1.66)	65.36 (1.34)	67.89 (1.39)	
Low HDL-C, % (SE)						<0.001
Yes	26.50 (0.01)	31.64 (1.41)	29.54 (1.54)	23.35 (1.47)	21.76 (1.33)	
No	73.50 (0.03)	68.36 (1.41)	70.46 (1.54)	76.65 (1.47)	78.24 (1.33)	
Elevated FPG, % (SE)						<0.001
Yes	25.41 (0.01)	27.06 (1.28)	28.73 (1.43)	26.05 (1.21)	20.01 (1.51)	
No	74.55 (0.03)	72.94 (1.28)	71.27 (1.43)	73.95 (1.21)	79.99 (1.51)	
Elevated WC, % (SE)						<0.001
Yes	55.79 (0.02)	64.37 (1.20)	60.32 (1.42)	55.07 (1.71)	43.98 (1.94)	
No	44.21 (0.02)	35.63 (1.20)	39.68 (1.42)	44.93 (1.71)	56.02 (1.94)	
Elevated BP, % (SE)						<0.001
Yes	31.95 (0.01)	35.58 (1.55)	35.52 (1.38)	30.10 (1.54)	26.79 (1.43)	
No	68.05 (0.03)	64.42 (1.55)	64.48 (1.38)	69.90 (1.54)	73.21 (1.43)	

The *p* value was calculated by the Kruskal-Wallis H test for continuous variables. The *p* value was calculated by Chi-square test for categorial variables. SE, standard error; MetS, metabolic syndrome; TG, triglycerides; HDL-C, high-density lipoprotein cholesterol; FPG, fasting plasma glucose; WC, waist circumference; BP, blood pressure.

### 3.2 Association between OBS and MetS

In the three models, the continuous OBS score was negatively correlated with MetS and its five components (all *p* < 0.001). The same correlation was observed between categorical OBS (divided by quartiles), MetS, and its five components (all *p*-values for trend <0.05). In fully adjusted model 3, OBS in Q3 and Q4 group was associated with lower odds of MetS compared to Q1 group (Q3 vs. Q1: odds ratio [OR] = 0.57, 95% confidence interval [CI], 0.46–0.71, *p* < 0.001; Q4 vs. Q1: OR = 0.42, 95%CI, 0.33–0.53, *p* < 0.001). Among the MetS components, participants in the Q3 and Q4 OBS groups demonstrated a higher risk of elevated TG, low HDL-C, elevated WC, and elevated BP than those in the Q1 group. Nevertheless, the inverse association between OBS in Q4 group, as opposed to Q2 and Q3 groups, and MetS was primarily identified in comparison to OBS in Q1 group, indicating that increasing OBS is associated with lower odds of MetS (OR = 0.64, 95% CI, 0.51–0.82, *p* < 0.001). The relationship between the OBS and MetS (including the five components) is shown in Table 2.

**Table 2:** Association of oxidative balance score with metabolic syndrome and its components.

	Model 1		Model 2		Model 3	
	OR (95%CI)	*p* value	OR (95%CI)	*p* value	OR (95%CI)	*p* value
MetS						
Continous OBS	0.96 (0.95,0.97)	<0.001	0.96 (0.95,0.97)	<0.001	0.95 (0.94,0.96)	<0.001
Quartile 1	Ref.		Ref.		Ref.	
Quartile 2	0.97 (0.83,1.13)	0.650	0.95 (0.80,1.12)	0.530	0.86 (0.72,1.04)	0.110
Quartile 3	0.69 (0.57,0.83)	<0.001	0.67 (0.55,0.82)	<0.001	0.57 (0,46,0.71)	<0.001
Quartile 4	0.53 (0.44,0.63)	<0.001	0.53 (0.44,0.65)	<0.001	0.42 (0.33,0.53)	<0.001
P for trend	<0.001		<0.001		<0.001	
Elevated TG						
Continous OBS	0.98 (0.97,0.99)	<0.001	0.98 (0.97,0.99)	<0.001	0.96 (0.95,0.97)	<0.001
Quartile 1	Ref.		Ref.		Ref.	
Quartile 2	0.92 (0.78,1.09)	0.630	0.99 (0.83,1.18)	0.900	0.90 (0.75,1.07)	0.230
Quartile 3	0.65 (0.53,0.79)	0.020	0.79 (0.67,0.94)	0.010	0.67 (0.55,0.80)	<0.001
Quartile 4	0.51 (0.42,0.62)	0.001	0.70 (0.58,0.84)	<0.001	0.54 (0.44,0.67)	<0.001
P for trend	<0.001			0.002	<0.001	
Low HDL-C						
Continous OBS	0.97 (0.96,0.97)	<0.001	0.97 (0.96,0.98)	<0.001	0.96 (0.95,0.97)	<0.001
Quartile 1	Ref.		Ref.		Ref.	
Quartile 2	0.91 (0.74,1.10)	0.320	0.92 (0.76,1.13)	0.430	0.87 (0.72,1.06)	0.870
Quartile 3	0.66 (0.55,0.79)	<0.001	0.67 (0.55,0.80)	<0.001	0.60 (0.50,0.73)	<0.001
Quartile 4	0.60 (0.49,0.74)	<0.001	0.61 (0.99,1.00)	<0.001	0.52 (0.41,0.67)	<0.001
P for trend	0.039		0.040		0.044	
Elevated FPG						
Continous OBS	0.98 (0.97,0.99)	<0.001	0.98 (0.97,0.99)	<0.001	0.98 (0.96,0.99)	<0.001
Quartile 1	Ref.		Ref.		Ref.	
Quartile 2	1.09 (0.93,1.27)	0.290	1.08 (0.91,1.27)	0.380	1.04 (0.88,1.24)	0.640
Quartile 3	0.95 (0.78,1.15)	0.590	0.97 (0.80,1.19)	0.790	0.91 (0.74,1.13)	0.400
Quartile 4	0.67 (0.55,0.83)	<0.001	0.70 (0.58,0.86)	<0.001	0.64 (0.51,0.82)	<0.001
P for trend	<0.001		0.001		0.001	
Elevated WC						
Continous OBS	0.95 (0.94,0.96)	<0.001	0.95 (0.94,0.96)	<0.001	0.93 (0.92,0.94)	<0.001
Quartile 1	Ref.		Ref.		Ref.	
Quartile 2	0.84 (0.73,0.97)	0.020	0.81 (0.70,0.95)	0.010	0.73 (0.62,0.86)	<0.001
Quartile 3	0.68 (0.59,0.78)	<0.001	0.63 (0.54,0.73)	<0.001	0.52 (0.44,0.62)	<0.001
Quartile 4	0.43 (0.36,0.52)	<0.001	0.40 (0.33,0.48)	<0.001	0.31 (0.25,0.38)	<0.001
P for trend	<0.001		<0.001		<0.001	
Elevated BP						
Continous OBS	0.98 (0.97,0.99)	<0.001	0.98 (0.97,0.99)	<0.001	0.97 (0.96,0.99)	<0.001
Quartile 1	Ref.		Ref.		Ref.	
Quartile 2	1.00 (0.84,1.19)	>0.999	0.97 (0.90,1.17)	0.010	0.94 (0.77,1.14)	0.500
Quartile 3	0.78 (0.65,0.93)	0.010	0.75 (0.62,0.92)	0.002	0.71 (0.57,0.90)	0.005
Quartile 4	0.66 (0.54,0.82)	<0.001	0.68 (0.53,0.86)	<0.001	0.62 (0.47,0.83)	0.002
P for trend	0.002		0.016		0.024	

MetS, metabolic syndrome; OBS, oxidative balance score; TG, triglycerides; HDL-C, high-density lipoprotein cholesterol; FPG, fasting plasma glucose; WC, waist circumference; BP, blood pressure.

**Figure 1:**
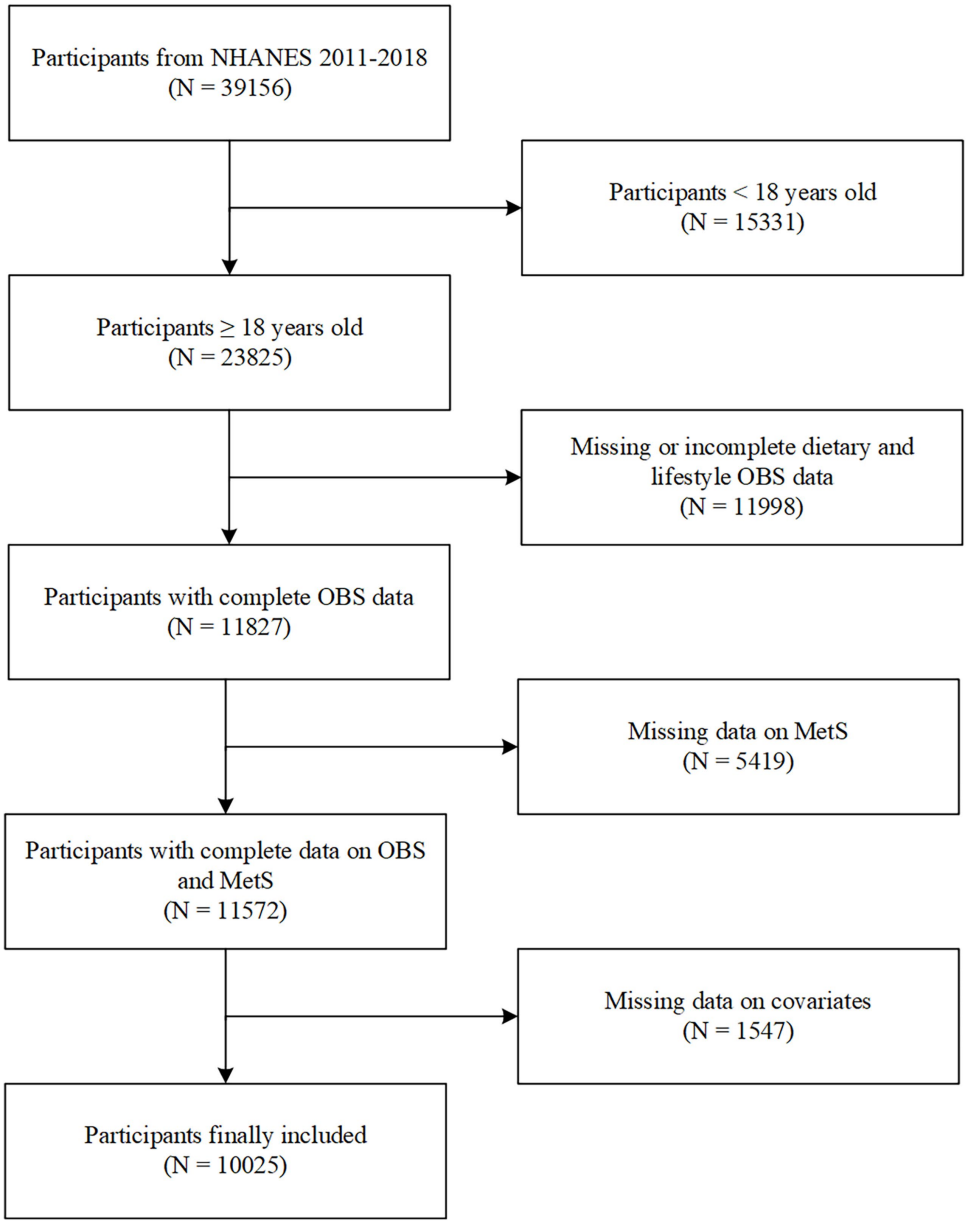
Flowchart of participants selection. NHANES, National Health and Nutrition Examination Survey; OBS, oxidative balance score; MetS, metabolic syndrome.

The associations among lifestyle, dietary OBS, and MetS are shown in Table 3. As continuous variables, dietary OBS and lifestyle OBS were significantly correlated with decreased risk of MetS (continuous dietary OBS: OR = 0.97, 95% CI, 0.96–0.98, *p* < 0.001; continuous lifestyle OBS: OR = 0.61, 95% CI, 0.58–0.64, p < 0.001) in fully adjusted model 3. In all models, individuals in dietary OBS Q3 and Q4 showed a higher risk of MetS than those in dietary OBS Q1. Lifestyle OBS scores in Q2, Q3, and Q4 demonstrated an inverse association with lifestyle OBS scores in Q1 in all three models (all *p* < 0.001). A low correlation between dietary and lifestyle OBS was observed (*r* value = 0.167) (see Figure 1).

**Table 3:** Association of dietary and lifestyle OBS with MetS.

	Model 1		Model 2		Model 3	
	OR (95%CI)	*p* value	OR (95%CI)	*p* value	OR (95%CI)	*p* value
Dietary OBS						
Continous dietary OBS	0.98 (0.97,0.99)	<0.001	0.98 (0.97,0.99)	<0.001	0.97 (0.96,0.98)	<0.001
OBS dietary quartile						
Q1	Ref.		Ref.		Ref.	
Q2	1.01 (0.86,1.19)	0.920	1.00 (0.84,1.19)	>0.999	0.93 (0.77,1.11)	0.400
Q3	0.73 (0.60,0.89)	0.003	0.74 (0.60,0.91)	0.010	0.66 (0.53,0.83)	<0.001
Q4	0.70 (0.59,0.84)	<0.001	0.72 (0.59,0.87)	0.001	0.60 (0.48,0.74)	<0.001
P for trend	<0.001		<0.001		<0.001	
Lifestyle OBS						
Continous lifestyle OBS	0.65 (0.62,0.68)	<0.001	0.61 (0.58,0.64)	<0.001	0.61 (0.58,0.64)	<0.001
Lifestyle OBS quartile						
Q1	Ref.		Ref.		Ref.	
Q2	0.66 (0.57,0.77)	<0.001	0.60 (0.51,0.71)	<0.001	0.60 (0.51,0.73)	<0.001
Q3	0.57 (0.48,0.67)	<0.001	0.74 (0.60,0.91)	<0.001	0.49 (0.41,0.59)	<0.001
Q4	0.14 (0.11,0.18)	<0.001	0.11 (0.09,0.15)	<0.001	0.11 (0.09,0.15)	<0.001
P for trend	<0.001		<0.001		<0.001	

MetS, metabolic syndrome; OBS, oxidative balance score.

### 3.3 Subgroup analysis

**Figure 2:**
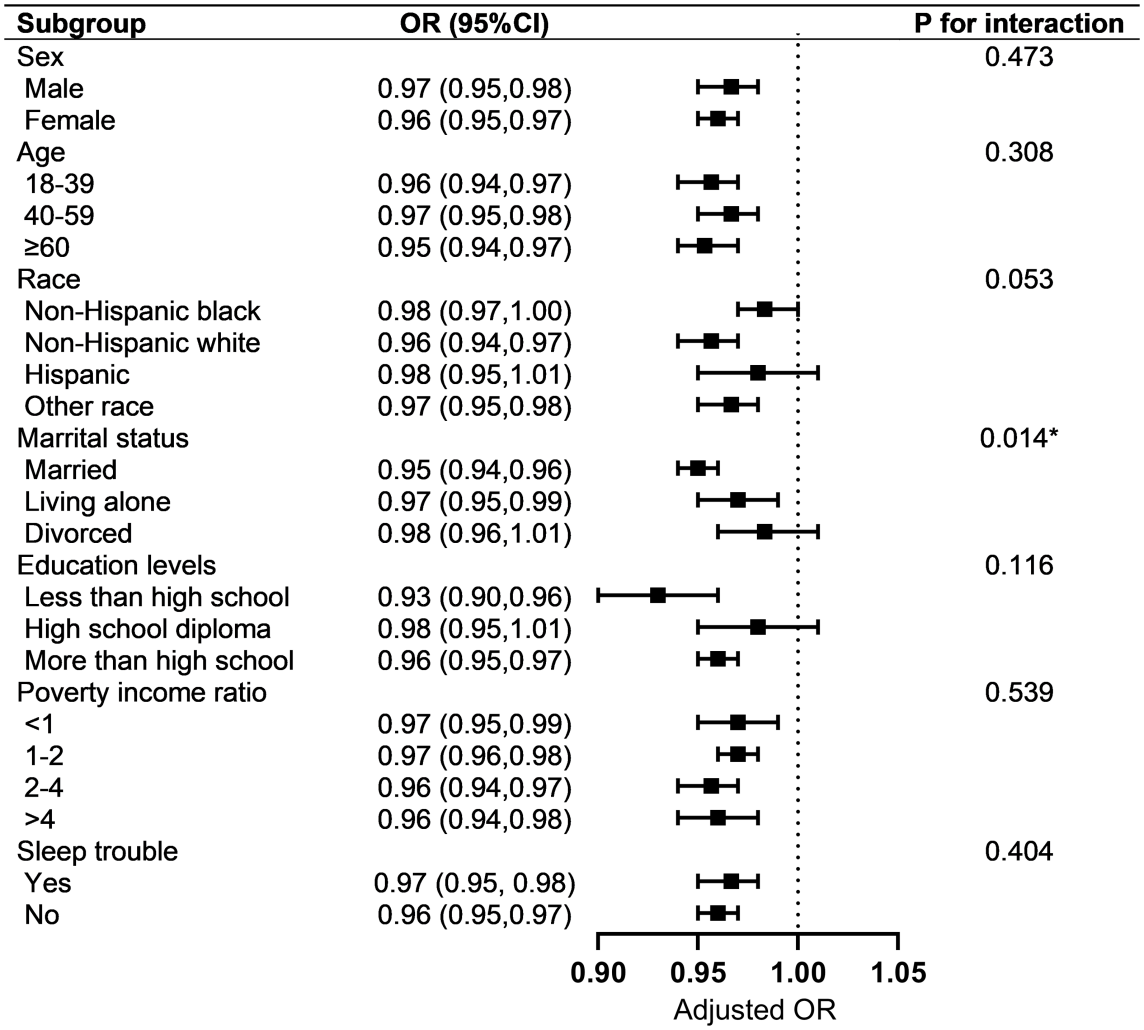
Subgroup analysis of the association between OBS and MetS. Each strata were adjusted for sex, age, race, marital status, education level, poverty income rate, and sleep problems. OBS, oxidative balance score; MetS, metabolic syndrome.

Subgroup analysis showed the association between OBS and MetS was not consistent among the different groups (Figure 2). Participants who were 18–39 years old, 40–59 years old, ≥ 60 years old, non-Hispanic black, non-Hispanic white, other race, male, female, married, living alone, less than high school, more than high school, with sleep trouble, without sleep trouble, PIR < 1, 1 ≤ PIR <2, 2 ≤ PIR ≤ 4, and PIR >4 demonstrated significant difference in subgroups stratified by age, sex, race, marital status, education levels, and sleep trouble (all *p* < 0.05). Meanwhile, a negative relationship was observed in individuals who were Hispanics, divorced, or had high school diplomas. However, this difference was not statistically significant (all *p* > 0.05). The characteristics, OBS (including total, dietary, and lifestyle OBS), and MetS (and its components) in the different subgroups are shown in Supplementary Tables S2–S8.

In the analysis of the interaction effect on subgroups, only marital status showed an interaction with the correlation between the OBS and MetS (*p* = 0.014). Interestingly, participants who were married showed less risk of MetS (OR = 0.95, 95% CI, 0.94–0.61, *p* < 0.001) compared to those who were living alone or divorced.

### 3.4 Analysis of restricted cubic spline regression

**Figure 3:**
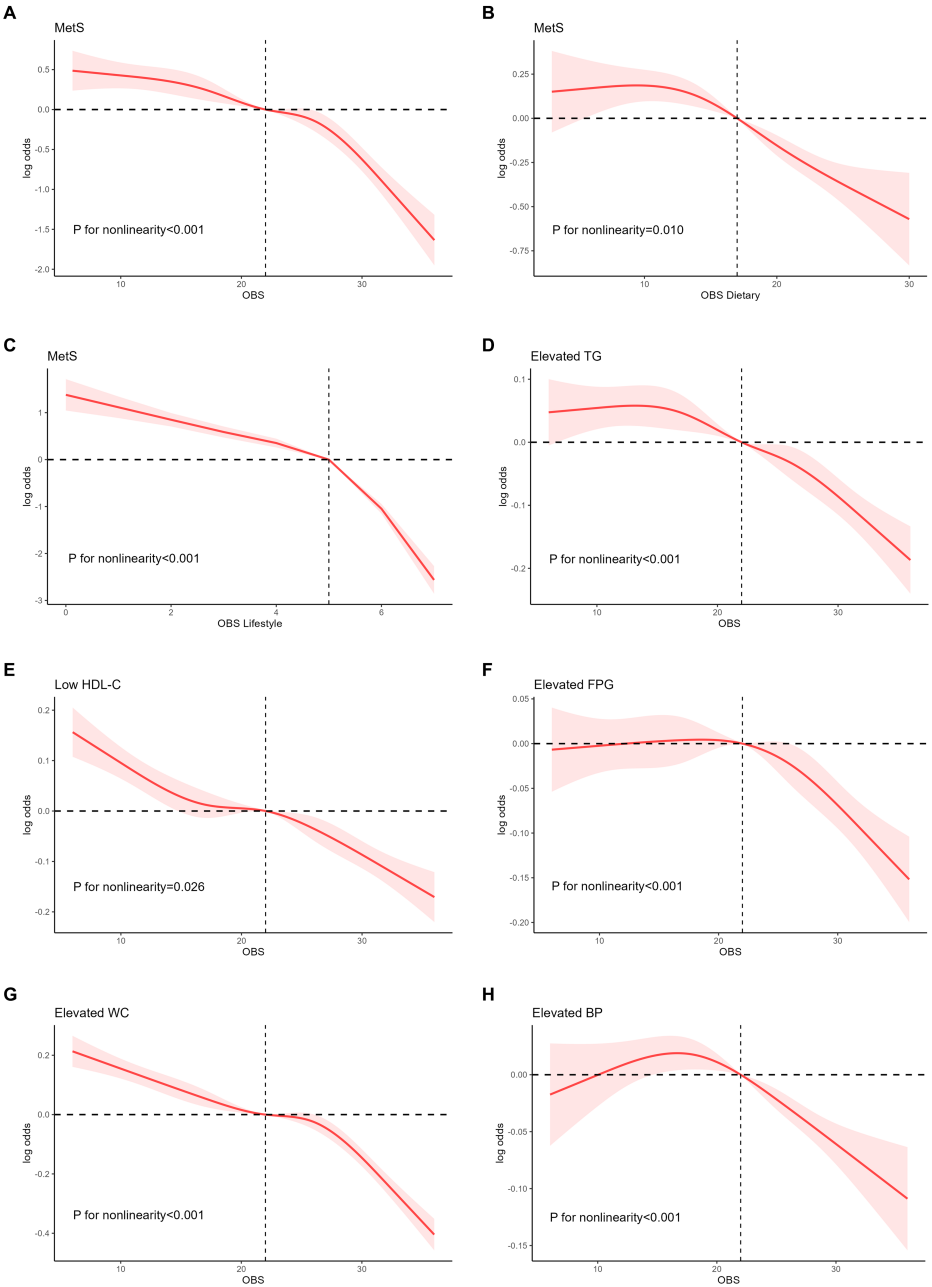
Nonlinear relationship of all OBS with MetS. **(A)** Nonlinear relationship between OBS and MetS. **(B)** Nonlinear relationship between dietary OBS and MetS. **(C)** Nonlinear relationship between lifestyle OBS and MetS. **(D)** Nonlinear relationship between OBS and elevated TG. **(E)** Nonlinear relationship between OBS and low HDL-C. **(F)** Nonlinear relationship between OBS and elevated FPG. **(G)** Nonlinear relationship between OBS and elevated WC. **(H)** Nonlinear relationship between OBS and elevated BP. OBS, oxidative balance score; MetS, metabolic syndrome; TG, triglyceride; HDL-C, high density cholesterols; WC, waist circumference; BP, blood pressure.

An analysis of the RCS regression is shown in Figure 3. After adjusting for all covariates, we found a significant nonlinear correlation between OBS (including dietary and lifestyle OBS) and MetS in the RCS regression (all *p* < 0.001).

After assessing RCS regression between OBS and five specific components of MetS, OBS was shown not obviously inverted U-shape correlation with elevated TG and elevated BP (all nonlinear *p* < 0.001), with 9 and 18 as the reflection point, respectively. Similarly, after the reflection point, a high risk of these components was observed (log OR above 0) and the risk started 218 to decrease. OBS was negatively related to low HDL-C and elevated WC (all nonlinear *p* < 0.001).

Supplementary Figure S1 demonstrated the nonlinear relationship between dietary OBS and five specific components of MetS. Nonlinear correlation was demonstrated between dietary OBS and elevated TG, elevated FPG and elevated BP (all *p* < 0.05). Nonlinear correlation between lifestyle OBS and five MetS components was shown in Supplementary Figure S2 (all *p* < 0.05).

## 4 Discussion

With the prevalence of Western lifestyles and economic growth, MetS, a new noncommunicable disease, has become a global health concern. A series of complex interacting factors lead to the pathogenesis of MetS, including fetal programming, chronic inflammation, and dysregulation of the redox system (1). The predictive value of the OBS for metabolic diseases is increasingly being recognized owing to the critical role of oxidative stress in metabolic disorders. To the best of our knowledge, this is the first large population-based study of adults in the US States to explore the correlation between OBS and MetS. This study provides concrete evidence of the negative association between OBS (total, dietary, and lifestyle OBS) and MetS and its components. Management of lifestyle and dietary factors may reduce the prevalence of MetS. Consistent with the findings of the total OBS, a significantly inverse correlation between dietary OBS and lifestyle OBS and MetS was observed. These findings suggest that improvements in lifestyle and dietary factors may effectively reduce the risk of developing MetS. However, in all models, the OR for lifestyle OBS was lower than that for dietary OBS, suggesting that the management of lifestyle choices may yield more benefits in reducing MetS risk.

The main components of MetS include abdominal obesity, hypertension, hypertriglyceridemia, low high-density lipoprotein cholesterol (HDL-C) levels, and increased FBG (33). Notably, these five factors are interrelated and interconnected. To further investigate the association between OBS and MetS, weighted logistic regression analyses using crude and adjusted models were conducted. The major finding was that OBS showed a strong positive correlation with TG, FBG, WC, and hypertension and a negative correlation with HDL-C. The relationship between obesity and oxidative stress has been widely studied (34). Nevertheless, the current literature offers limited evidence of a connection between OBS and WC. A cross-sectional study in Iran has presented evidence indicating a negative association between OBS and WC (OR = 0.55, 95% CI, 0.38–0.81, *p* = 0.003), which aligns with our own findings (2). The bidirectional effect between obesity and oxidative stress may be responsible, wherein obesity can contribute to, and be a consequence of oxidative stress (35). *In vitro* cell culture and animal studies have demonstrated that oxidative stress can stimulate the proliferation of pre-adipocytes, promote adipocyte differentiation, and enlarge mature adipocytes (36). Furthermore, research has revealed that ROS elicit diverse effects on hypothalamic neurons responsible for the regulation of satiety and hunger behavior, thereby exerting an influence on the mechanisms involved in weight control (37) and can induce increased generation of ROS, leading to oxidative stress through various pathways, ultimately establishing a detrimental cycle (3).

A previous cross-sectional study conducted in racially and ethnically diverse populations suggested an inverse connection between OBS and hypertension after accounting for covariates (23). Furthermore, a prospective cohort study based on community, which included 5,181 participants showed that individuals with high OBS exhibited a reduced risk of developing hypertension (38). This negative relationship is consistent with the trend observed in the present study. The underlying mechanisms may involve endothelial damage, vascular dysfunction, renal impairment, hyperactivity of the sympathetic nervous system (SNS), and disturbances in the renin-angiotensin-aldosterone (RAAS) induced by oxidative stress, ultimately leading to the development of hypertension (39, 40).

This study found that higher OBS values were associated with a decreased risk of elevated FBG (24). Oxidative stress is involved in the onset and progression of diabetes and IR (14). Similarly, an inverse association between OBS and type 2 diabetes mellitus was demonstrated in a study involving 7,369 participants (41). Damage to mitochondria plays a pivotal role in this process, which involves the generation of mitochondrial H_2_O_2_ and the activation of NOX (42). Oxidative stress triggers the activation of casein kinase-2 (CK2), which in turn activates the retromer. This altered retromer redirects glucose transporter 4 (GLUT4) from the plasma membrane to the trans-Golgi network for transportation to the lysosomes, resulting in the destruction of GLUT4. Ultimately, this cascade of events contributes to the development of hyperglycemia (43).

Currently, the association between oxidative stress and LDL-C levels has been widely discussed (7, 44, 45). However, the potential correlation between the OBS and TG and HDL-C levels is debatable. A cross-sectional study involving 847 participants in Tehran revealed no significant correlation between the OBS and TG and HDL-C levels. In this study conducted in Iran, the OBS was constructed using a selection of 13 dietary and lifestyle components, while alcohol drinking was missing in the OBS. These differences may have contributed to the disparities observed between our findings and those of previous research results (2).

We speculate that this correlation is associated with mitochondrial dysfunction caused by oxidative stress. This dysfunction disrupts mitochondrial utilization of lipids, leading to lipid accumulation in the tissues (46). It has also been hypothesized that the tricarboxylic cycle enzyme aconitase can be inhibited by increased superoxide levels, thus favoring citrate accumulation, which is used as a substrate for the production of fatty acids and cholesterol (46). Excessive fatty acids are directed to the liver, resulting in the increased synthesis of very low-density lipoprotein (VLDL). This leads to high TG levels in the bloodstream and the exchange of TG from VLDL for cholesterol in HDL, resulting in low HDL-C levels (47).

Through subgroup analysis and interaction testing, we found that the negative correlation between the OBS and MetS was inconsistent across the different subgroups. Factors, such as age, race, sex, education, sleep status, and poverty status, did not show a negative correlation between OBS and MetS. However, significant differences were observed according to marital status. In the married population, OBS had a significant impact on the prevalence of MetS (OR = 0.948, 95% CI, 0.935–0.961; *p* < 0.0001). These findings imply that enhancing dietary and lifestyle habits among married individuals may yield greater efficacy in preventing the occurrence of MetS. This may be associated with the influence of marital status on the endocrine and immune systems (48). Intimate relationships have been shown to cause physiological changes in the human body via emotional alterations. Marital conflicts occur regularly among married couples, averaging approximately one to two conflict discussions per month (48). During conflict, individuals show increased levels of epinephrine, norepinephrine, ACTH, growth hormone, and decreased levels of prolactin. The immune system exhibits increased NK cell lysis and blastogenic responses to mitogens (concanavalin A and PHA), as well as higher antibody levels against latent Epstein–Barr virus. Notably, females tend to exhibit greater changes in endocrine function and immunological responses than males (49). Further research is needed to investigate the potential relationship between the aforementioned influence and oxidative stress, as no studies on this association currently exist.

Furthermore, this study employed flexible modeling using the RCS to visually depict the dose–response relationship between OBS and MetS. As the OBS increased, the risk of MetS gradually decreased, and this correlation became more pronounced at higher OBS levels. The activation of receptors, such as nod-like receptor family pyrin domain containing 3 (3) and peroxisome proliferator-activated receptor-γ (50) assumes a critical function in the occurrence of oxidative stress and MetS. In biological systems, receptor-mediated responses typically exhibit strong dose-dependence, followed by a plateau phase in which the response no longer increases with further dose escalation (32). This phenomenon may account for the more pronounced downward trend observed. The association between dietary OBS and MetS exhibited an inverted U-shaped pattern, with a gradual increase until a threshold OBS of 9.51. Beyond this threshold, the risk of MetS decreased substantially as OBS levels increased. Furthermore, analogous inverted U-shaped associations have been identified in various OBS components, such as elevated TG, FPG, and hypertension.

This study had several key advantages. First, our study identified, for the first time, the association between OBS and MetS as well as MetS components in the US population. In addition, we examined the association between dietary OBS, lifestyle OBS, and MetS. Second, the NHANES employed a stratified, multistage sampling methodology, which increased the external validity of our study results by ensuring representation of the non-institutionalized population. Third, the use of complex statistical methods ensures a comprehensive and reliable outcome. This study established sophisticated models that accounted for multiple confounding factors, and the OBS scores were adjusted to accommodate both continuous and categorical variables to mitigate any potential effects on the observed associations. Furthermore, subgroup analyses were performed to explore the potential influence of other factors on the association between OBS and MetS.

However, this study has several limitations that should be acknowledged. First, cross-sectional studies have limitations in terms of establishing causal relationships, necessitating additional prospective studies. Second, we could not completely exclude the potential influence of all confounding factors. Third, the exclusion of individuals with missing data may have introduced a selection bias. Finally, information regarding dietary components was acquired using 24-h food recalls, which are known to have limitations in accurately determining absolute nutrient values. Additionally, the data collected pertaining to the average intake over the previous year may be susceptible to recall bias.

Despite these limitations, this study has significant clinical implications. While certain factors in the causal relationship with MetS cannot be altered, many can be corrected and attenuated. By integrating the influences of diet and lifestyle, OBS provides a more comprehensive approach to assess oxidation–reduction status. Through OBS assessment, individuals at a high risk of MetS can be identified at an early stage. Furthermore, we can effectively prevent and protect individuals at high risk of MetS while providing valuable treatment guidance for individuals diagnosed with MetS by implementing OBS-enhancing strategies, including consuming antioxidant-rich foods, incorporating regular exercise, and quitting smoking.

## 5 Conclusion

In conclusion, this study suggests a strong association between the OBS and MetS and its components. Our data indicate that a higher OBS, as well as dietary and lifestyle OBS, are correlated with a decreased risk of MetS and its components in a nonlinear manner. Hence, the OBS may serve as an effective marker for identifying individuals with MetS, with a higher score indicating a predominance of more antioxidants. However, further multicenter prospective cohort studies are required to validate our findings.

## Data availability statement

Publicly available datasets were analyzed in this study. This data can be found here: Centers for Disease Control and Prevention (CDC), National Center for Health Statistics (NCHS), National Health and Nutrition Examination Survey (NHANES), https://wwwn.cdc.gov/nchs/nhanes/.

## Ethics statement

The studies involving humans were approved by The National Centre for Health Statistics' Ethical Review Committee. The studies were conducted in accordance with the local legislation and institutional requirements. The participants provided their written informed consent to participate in this study.

## Author contributions

YL: Conceptualization, Writing – original draft. MW: Supervision, Writing – review & editing. JB: Methodology, Writing – review & editing. DC: Writing – review & editing. HJ: Investigation, Methodology, Writing – review & editing.
