# Supplementary figures and images for: Association between oxidative balance score and metabolic syndrome and its components in US adults: a cross-sectional study from NHANES 2011–2018
Source: Front Nutr. 2024 Mar 13;11:1375060. doi: 10.3389/fnut.2024.1375060 (PMC10966126; doi:10.3389/fnut.2024.1375060)

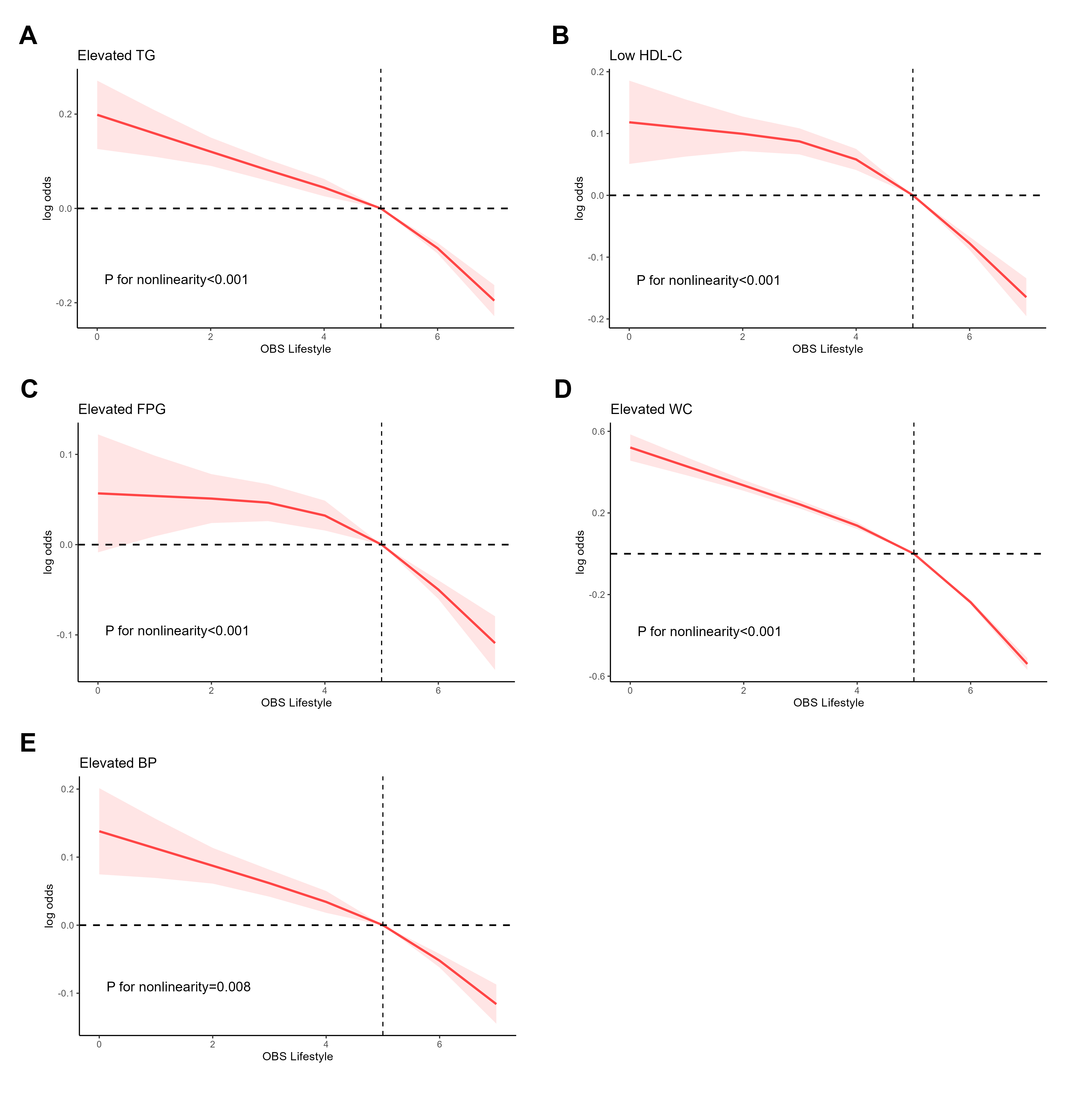

Supplement: Supplementary file 3 [file Image_2.TIF]
