# Supplementary material for: Association between oxidative balance score and metabolic syndrome and its components in US adults: a cross-sectional study from NHANES 2011–2018
Source: Front Nutr. 2024 Mar 13;11:1375060. doi: 10.3389/fnut.2024.1375060 (PMC10966126; doi:10.3389/fnut.2024.1375060)
Supplement: Supplementary file 4 [file Table_1.DOCX]

Association between oxidative balance score and metabolic syndrome and its components in US adults: A cross-section study from NHANES 2011-2018

Yi Lu^1^, Meixiang Wang^1^, Jiaxin Bao^2^, Dashuang Chen^3^, Hao Jiang^1*^

^1^Department of Cardiology, The Affiliated Taizhou People’s Hospital of Nanjing Medical University, Taizhou School of Clinical Medicine, Nanjing Medical University, Taizhou 225300, China

^2^Department of Nephrology, The Affiliated Taizhou People’s Hospital of Nanjing Medical University, Taizhou School of Clinical Medicine, Nanjing Medical University, Taizhou 225300, China

^3^Department of General Medicine, The Affiliated Taizhou People’s Hospital of Nanjing Medical University, Taizhou School of Clinical Medicine, Nanjing Medical University, Taizhou 225300, China

*** Correspondence:**Hao Jiang, M.D.
jianghao555666@163.com

**Keywords: Oxidative balance score (OBS), metabolic syndrome, NHANES, oxidative stress, antioxidant**

**Supplementary Appendix**

This appendix has been provided by the authors to give readers additional information about their work.

**List of Contents**

Page 1: Cover page

Page 2: List of contents

Page 3: Supplemental Table S1

Page 4-5: Supplemental Table S2

Page 6-7: Supplemental Table S3

Page 8-9: Supplemental Table S4

Page 10-11: Supplemental Table S5

Page 12-13: Supplemental Table S6

Page 14-15: Supplemental Table S7

Page 16-17: Supplemental Table S8

Page 18: Supplemental Figure 1

Page 19: Supplemental Figure 2

| OBS components | Assignment scheme | | |
| --- | --- | --- | --- |
|  | 0 | 1 | 2 |
| Lifestyle prooxidants |  |  |  |
| Alcohol (g/d) | ≥30 for male  ≥15 for female | 0-30 for male 0-15 for female | None |
| Body mass index (kg/m2) | >30 | 25-30 | <25 |
| Cotinine (ng/mL) | >0.038 | 0.038-1.13 | <1.13 |
| Lifestyle antioxidants |  |  |  |
| Physical activity (MET-minute/week) | <400 | 400-1,000 | >1,000 |
| Dietary prooxidants |  |  |  |
| Total fat (g/d) | >107.43 | 69.83-107.43 | <69.83 |
| Iron (mg/d) | >19.17 | 12.88-19.17 | <12.88 |
| Dietary antioxidants |  |  |  |
| Dietary fiber (g/d) | <12.56 | 12.56-19.70 | >19.70 |
| Carotene (RE/d) | <98.83 | 98.83-306.25 | >306.25 |
| Riboflavin (mg/d) | <1.79 | 1.79-2.69 | >2.69 |
| Niacin (mg/d) | <20.65 | 20.65-29.75 | >29.75 |
| Total folate (mcg/d) | <316.00 | 316.00-492.00 | >492.00 |
| Vitamin B6 (mg/d) | <1.59 | 1.59-2.40 | >2.40 |
| Vitamin B12 (mcg/d) | <3.36 | 3.36-6.20 | >6.20 |
| Vitamin C (mg/d) | <42.44 | 42.44-113.21 | >113.21 |
| Vitamin E (ATE) (mg/d) | <5.82 | 5.82-9.42 | >9.42 |
| Calcium (mg/d) | <646.00 | 646.00-1072.00 | >1072 |
| Magnesium (mg/d) | <257.00 | 257.00-361.28 | >361.28 |
| Zinc (mg/d) | <9.75 | 9.75-15.10 | >15.10 |
| Copper (mg/d) | <1.12 | 1.12-1.57 | >1.57 |
| Selenium (mcg/d) | <94.94 | 94.94-141.80 | >141.80 |

**Supplemental Table S1** Assignment scheme of Oxidative Balance Score.
OBS, oxidative balance score; RE, retinol equivalent; ATE, alpha-tocopherol; MET, metabolic equivalent.

**Supplemental Table S2** Baseline characteristics of the participants stratified by gender.

| Variable | Male | Female | P value |
| --- | --- | --- | --- |
| Age (years) | 46 (31,59) | 48 (33,60) | **<0.001** |
| Race/Ethnicity, % (SE) |  |  | 0.120 |
| Non-Hispanic Black | 9.38 (0.84) | 10.30 (1.01) |  |
| Non-Hispanic White | 68.51 (1.74) | 69.23 (1.84) |  |
| Hispanic | 5.45 (0.54) | 5.20 (0.55) |  |
| Other race | 16.66 (1.05) | 15.27 (1.01) |  |
| Education levels, % (SE) |  |  | 0.250 |
| Less than high school | 2.78 (0.27) | 2.35 (0.28) |  |
| High school diploma | 7.51 (0.63) | 6.92 (0.66) |  |
| More than high school | 89.70 (0.77) | 90.73 (0.77) |  |
| Marital status, % (SE) |  |  | **<0.001** |
| Married | 56.57 (1.35) | 53.70 (1.22) |  |
| Divorced | 8.42 (0.64) | 12.62 (0.64) |  |
| Living alone | 35.01 (1.35) | 33.68 (1.25) |  |
| Poverty income ratio | 3.33 (1.70,5.00) | 3.17 (1.57,5.00) | **0.010** |
| Energy (kcal) | 2391 (1820,3065) | 1757 (1340,2222) | **<0.001** |
| Caffeine (mg) | 144 (36,278) | 109 (28,223) | **<0.001** |
| Sleep trouble, % (SE) |  |  | **<0.001** |
| Yes | 25.33 (0.96) | 33.05 (1.07) |  |
| No | 74.67 (0.96) | 66.95 (1.07) |  |
| MetS, % (SE) |  |  | 0.710 |
| Yes | 29.77 (0.91) | 30.20 (1.15) |  |
| No | 70.23 (0.91) | 69.80 (1.15) |  |
| Elevated TG, % (SE) |  |  | **<0.001** |
| Yes | 42.43 (1.28) | 29.82 (1.09) |  |
| No | 57.57 (1.28) | 70.18 (1.09) |  |
| Low HDL-C, % (SE) |  |  | **<0.001** |
| Yes | 23.17 (0.94) | 30.03 (1.05) |  |
| No | 76.83 (0.94) | 69.97 (1.05) |  |
| Elevated FPG, % (SE) |  |  | **<0.001** |
| Yes | 29.61 (1.11) | 21.04 (0.74) |  |
| No | 70.39 (1.11) | 78.96 (0.74) |  |
| Elevated WC, % (SE) |  |  | **<0.001** |
| Yes | 46.29 (1.16) | 65.88 (1.27) |  |
| No | 53.71 (1.16) | 34.12 (1.27) |  |
| Elevated BP, % (SE) |  |  | **0.010** |
| Yes | 33.18 (1.06) | 30.65 (0.86) |  |
| No | 66.82 (1.06) | 69.35 (0.86) |  |
| OBS | 22 (17,27) | 23 (17,28) | **0.020** |
| Dietary OBS | 18 (12,23) | 18 (13,23) | **0.040** |
| Lifestyle OBS | 5 (4,6) | 5 (4,6) | 0.220 |

The P value was calculated by the Kruskal-Wallis H test for continuous variables. The P value was calculated by Chi-square test for categorial variables. SE, standard error; MetS, metabolic syndrome; TG, triglycerides; HDL-C, high-density lipoprotein cholesterol; FPG, fasting plasma glucose; WC, waist circumference; BP, blood pressure; OBS, oxidative balance score.

**Supplemental Table S3** Baseline characteristics of the participants stratified by age.

| Variable | 18-39 | 40-59 | ≥60 | P value |
| --- | --- | --- | --- | --- |
| Gender, % (SE) |  |  |  | **0.001** |
| Male | 54.11 (0.80) | 50.86 (1.15) | 48.44 (0.88) |  |
| Female | 45.89 (0.80) | 49.14 (1.15) | 51.56 (0.88) |  |
| Race/Ethnicity, % (SE) |  |  |  | **<0.001** |
| Non-Hispanic Black | 11.47 (1.09) | 10.05 (0.97) | 6.97 (0.74) |  |
| Non-Hispanic White | 59.79 (2.06) | 69.62 (1.91) | 81.49 (1.37) |  |
| Hispanic | 7.15 (0.65) | 4.85 (0.58) | 3.31 (0.48) |  |
| Other race | 21.60 (1.29) | 15.49 (1.14) | 8.23 (0.67) |  |
| Education levels, % (SE) |  |  |  | 0.090 |
| Less than high school | 2.08 (0.36) | 2.52 (0.32) | 3.42 (0.40) |  |
| High school diploma | 7.6 (0.68） | 7.19 (0.72) | 6.69 (0.73) |  |
| More than high school | 90.32 (0.82) | 90.29 (0.92) | 89.89 (0.91) |  |
| Marital status, % (SE) |  |  |  | **<0.001** |
| Married | 39.27 (1.52) | 63.59 (1.54) | 66.30 (1.55) |  |
| Divorced | 3.52 (0.37) | 15.88 (0.95) | 12.59 (0.84) |  |
| Living alone | 57.21 (1.64) | 20.53 (1.21) | 21.10 (1.28) |  |
| Poverty income ratio | 2.55 (1.23,4.49) | 3.71 (1.95,5.00) | 3.67 (2.02,5.00) | **<0.001** |
| Energy (kcal) | 2159 (1586,2822) | 2106 (1580,2722) | 1848 (1412,2390) | **<0.001** |
| Caffeine (mg) | 86 (6,192) | 156 (59,288) | 144 (43,264) | **<0.001** |
| Sleep trouble, % (SE) |  |  |  | **<0.001** |
| Yes | 79.05 (1.09) | 67.24 (1.19) | 64.29 (1.18) |  |
| No | 20.95 (1.09) | 32.76 (1.19) | 35.71 (1.18) |  |
| MetS, % (SE) |  |  |  | **<0.001** |
| Yes | 16.10 (0.99) | 34.88 (1.17) | 43.38 (1.68) |  |
| No | 83.90 (0.99) | 65.12 (1.17) | 56.52 (1.68) |  |
| Elevated TG, % (SE) |  |  |  | **<0.001** |
| Yes | 28.82 (1.23) | 41.41 (1.25) | 39.79 (1.46) |  |
| No | 71.18 (1.23) | 58.89 (1.25) | 60.21 (1.46) |  |
| Low HDL-C, % (SE) |  |  |  | **0.001** |
| Yes | 27.82 (1.08) | 27.83 (1.27) | 22.42 (1.13) |  |
| No | 72.18 (1.08) | 72.17 (1.27) | 77.58 (1.13) |  |
| Elevated FPG, % (SE) |  |  |  | **<0.001** |
| Yes | 14.49 (0.91) | 27.36 (1.17( | 39.16 (1.60) |  |
| No | 85.51 (0.91) | 72.64 (1.17) | 60.84 (1.60) |  |
| Elevated WC, % (SE) |  |  |  | **<0.001** |
| Yes | 43.11 (1.40) | 59.99 (1.27) | 68.58 (1.67) |  |
| No | 56.89 (1.40) | 40.01 (1.27) | 31.42 (1.67) |  |
| Elevated BP, % (SE) |  |  |  | **<0.001** |
| Yes | 8.90 (0.68) | 35.08 (1.38) | 62.15 (1.59) |  |
| No | 91.10 (0.68) | 64.92 (1.38) | 37.85 (1.59) |  |
| OBS | 23 (13,28) | 23 (17,28) | 22 (17,27) | 0.460 |
| Dietary OBS | 18 (12,23) | 18 (13,23) | 17 (12,22) | 0.160 |
| Lifestyle OBS | 5 (4,6) | 5 (4,6) | 5 (4,6) | <0.001 |

The P value was calculated by the Kruskal-Wallis H test for continuous variables. The P value was calculated by Chi-square test for categorial variables. SE, standard error; MetS, metabolic syndrome; TG, triglycerides; HDL-C, high-density lipoprotein cholesterol; FPG, fasting plasma glucose; WC, waist circumference; BP, blood pressure; OBS, oxidative balance score.

**Supplemental Table S4** Baseline characteristics of the participants stratified by race/ethnicity.

| Variable | Non-Hispanic Black | Non-Hispanic White | Hispanic | Other race | P value |
| --- | --- | --- | --- | --- | --- |
| Age (years) | 43 (30,55) | 50 (34,62) | 39 (29,53) | 39 (29,52) | **<0.001** |
| Gender, % (SE) |  |  |  |  | 0.120 |
| Male | 49.13 (1.20) | 51.21 (0.80) | 52.66 (1.94) | 53.65 (1.28) |  |
| Female | 50.87 (1.20) | 48.79 (0.80) | 47.34 (1.94) | 46.35 (1.28) |  |
| Education levels, % (SE) |  |  |  |  | **<0.001** |
| Less than high school | 2.17 (0.39) | 0.80 (0.16) | 8.54 (1.11) | 8.49 (0.95) |  |
| High school diploma | 10.84 (0.81) | 5.42 (0.68) | 13.24 (1.90) | 10.78 (0.87) |  |
| More than high school | 86.99 (0.97) | 93.79 (0.75) | 78.22 (2.25) | 80.73 (1.50) |  |
| Marital status, % (SE) |  |  |  |  | **<0.001** |
| Married | 31.32 (1.23) | 59.87 (1.22) | 46.43 (2.29) | 52.57 (1.95) |  |
| Divorced | 13.42 (1.01) | 10.75 (0.61) | 9.29 (1.15） | 7.78 (0.91) |  |
| Living alone | 55.27 (1.55) | 29.38 (1.20) | 44.29 (2.40) | 39.66 (1.75) |  |
| Poverty income ratio | 1.98 (0.99,3.66) | 3.74 (2.02,5.00) | 2.03 (1.08,3.69) | 2.36 (1.20,4.30) | **<0.001** |
| Energy (kcal) | 2001 (1465,2683) | 2068 (1551,2698) | 2002 (1470,2702) | 2057 (1532,2462) | 0.320 |
| Caffeine (mg) | 33 (0,117) | 153 (55,286) | 102 (23,198) | 88 (14,180) | **<0.001** |
| Sleep trouble, % (SE) |  |  |  |  | **<0.001** |
| Yes | 26.40 (1.66) | 31.85 (1.00) | 22.47 (1.79) | 20.97 (1.40) |  |
| No | 73.60 (1.66) | 68.15 (1.00) | 77.53 (1.79) | 79.03 (1.40) |  |
| MetS, % (SE) |  |  |  |  | 0.530 |
| Yes | 28.00 (1.25) | 30.40 (1.12) | 29.51 (2.25) | 29.53 (1.15) |  |
| No | 72.00 (1.25) | 69.60 (1.12) | 70.49 (2.25) | 70.47 (1.15) |  |
| Elevated TG, % (SE) |  |  |  |  | **<0.001** |
| Yes | 20.36 (1.07) | 36.96 (1.11) | 40.42 (2.16) | 41.95 (1.32) |  |
| No | 79.64 (1.07) | 63.04 (1.11) | 59.58 (2.16) | 58.05 (1.32) |  |
| Low HDL-C, % (SE) |  |  |  |  | **<0.001** |
| Yes | 25.64 (1.07) | 24.76 (0.90) | 32.52 (2.29) | 32.51 (1.42) |  |
| No | 74.36 (1.07) | 75.24 (0.90) | 67.48 (2.29) | 67.49 (1.42) |  |
| Elevated FPG, % (SE) |  |  |  |  | **0.540** |
| Yes | 24.23 (1.26) | 25.44 (0.99) | 24.15 (1.90) | 26.68 (1.23) |  |
| No | 75.77 (1.26) | 74.56 (0.99) | 75.85 (1.90) | 73.32 (1.23) |  |
| Elevated WC, % (SE) |  |  |  |  | **<0.001** |
| Yes | 60.74 (1.51) | 56.79 (1.28) | 52.43 (2.15) | 49.56 (1.72) |  |
| No | 39.26 (1.51) | 43.21 (1.28) | 47.57 (2.115) | 50.44 (1.72) |  |
| Elevated BP, % (SE) |  |  |  |  | **<0.001** |
| Yes | 40.53 (1.20) | 32.80 (1.10) | 23.21 (1.91） | 25.94 (1.00) |  |
| No | 59.47 (1.20) | 67.20 (1.10) | 76.79 (1.91) | 74.06 (1.00) |  |
| OBS | 19 (14,25) | 23 (17,28) | 23 (17,28) | 23 (18,28) | **<0.001** |
| Dietary OBS | 15 (10,21) | 18 (13,23) | 18 (12,23) | 18 (13,23) | **<0.001** |
| Lifestyle OBS | 4 (3,5) | 5 (4,6) | 5 (4,6) | 5 (4,6) | **<0.001** |

The P value was calculated by the Kruskal-Wallis H test for continuous variables. The P value was calculated by Chi-square test for categorial variables. SE, standard error; MetS, metabolic syndrome; TG, triglycerides; HDL-C, high-density lipoprotein cholesterol; FPG, fasting plasma glucose; WC, waist circumference; BP, blood pressure; OBS, oxidative balance score.

**Supplemental Table S5** Baseline characteristics of the participants stratified by marrital status.

| Variable | Married | Divorced | Living alone | P value |
| --- | --- | --- | --- | --- |
| Age (years) | 50 (39,62) | 55 (47,61) | 32 (24,51) | **<0.001** |
| Gender, % (SE) |  |  |  | **<0.001** |
| Male | 52.78 (0.66) | 41.45 (2.40) | 52.44 (1.06) |  |
| Female | 47.22 (0.66) | 58.55 (2.40) | 47.56 (1.06) |  |
| Race/Ethnicity, % (SE) |  |  |  | **<0.001** |
| Non-Hispanic Black | 16.05 (1.46) | 10.34 (1.06) | 7.91 (0.80) |  |
| Non-Hispanic White | 64.63 (2.18) | 64.63 (2.18) | 70.75 (2.02) |  |
| Hispanic | 4.48 (0.48) | 4.73 (0.64) | 6.87 (0.74) |  |
| Other race | 15.23 (1.09) | 11.89 (1.55) | 18.45 (1.27) |  |
| Education levels, % (SE) |  |  |  | **<0.001** |
| Less than high school | 2.42 (0.27) | 1.60 (0.31) | 3.12 (0.41) |  |
| High school diploma | 6.09 (0.56) | 7.95 (1.19) | 8.82 (0.77) |  |
| More than high school | 91.49 (0.70) | 90.46 (1.27) | 88.06 (0.94) |  |
| Poverty income ratio, % (SE) | 4.07 (2.33,5.00) | 2.43 (1.28,4.21) | 2.15 (1.07,4.21) | **<0.001** |
| Energy (kcal) | 2070 (1586,2663) | 1993 (1422,2674) | 2070 (1586,2663) | 0.080 |
| Caffeine (mg) | 144 (45,268) | 146 (58,302) | 96 (7,2010 | **<0.001** |
| Sleep trouble, % (SE) |  |  |  | **0.003** |
| Yes | 27.39 (0.92) | 35.82 (2.29) | 29.74 (1.45) |  |
| No | 72.61 (0.92) | 64.18 (2.29) | 70.26 (1.45) |  |
| MetS, % (SE) |  |  |  | **<0.001** |
| Yes | 32.52 (1.04) | 37.68 (2.17) | 23.56 (1.18) |  |
| No | 67.48 (1.04) | 62.32 (2.17) | 76.44 (1.18) |  |
| Elevated TG, % (SE) |  |  |  | **<0.001** |
| Yes | 38.85 (0.95) | 39.78 (2.52) | 31.17 (1.35) |  |
| No | 61.15 (0.95) | 60.22 (2.52) | 68.83 (1.35) |  |
| Low HDL-C, % (SE) |  |  |  | 0.510 |
| Yes | 26.86 (0.93) | 27.64 (2.09) | 25.57 (1.11) |  |
| No | 73.14 (0.93) | 72.36 (2.09) | 74.43 (1.11) |  |
| Elevated FPG, % (SE) |  |  |  | **<0.001** |
| Yes | 28.05 (1.12) | 30.50 (1.82) | 19.74 (1.04) |  |
| No | 71.95 (1.12) | 69.50 (1.82) | 80.26 (1.04) |  |
| Elevated WC, % (SE) |  |  |  | **<0.001** |
| Yes | 57.98 (1.28) | 65.92 (2.49) | 49.20 (1.46) |  |
| No | 42.02 (1.28) | 34.08 (2.49) | 50.80 (1.46) |  |
| Elevated BP, % (SE) |  |  |  | **<0.001** |
| Yes | 35.60 (1.08) | 39.49 (1.86) | 23.79 (1.21) |  |
| No | 64.40 (1.08) | 60.51 (1.86) | 76.21 (1.21) |  |
| OBS | 24 (18,28) | 21 (16,27) | 22 (16,27) | **<0.001** |
| Dietary OBS | 19 (13,23) | 17 (12,22) | 17 (11,22) | **<0.001** |
| Lifestyle OBS | 5 (4,6) | 4 (3,5) | 5 (3,5) | **<0.001** |

The P value was calculated by the Kruskal-Wallis H test for continuous variables. The P value was calculated by Chi-square test for categorial variables. SE, standard error; MetS, metabolic syndrome; TG, triglycerides; HDL-C, high-density lipoprotein cholesterol; FPG, fasting plasma glucose; WC, waist circumference; BP, blood pressure; OBS, oxidative balance score.

**Supplemental Table S6** Baseline characteristics of the participants stratified by educational levels.

| Variable | Less than high school | High school | More than high school | P value |
| --- | --- | --- | --- | --- |
| Age (years) | 50 (37,64） | 46 (32,58) | 47 (32,59) | **0.020** |
| Gender, % (SE) |  |  |  | 0.250 |
| Male | 55.65 (2.51) | 53.54 (2.44) | 51.19 (0.61) |  |
| Female | 44.35 (2.51) | 46.46 (2.44) | 48.81 (0.61) |  |
| Race/Ethnicity, % (SE) |  |  |  | **<0.001** |
| Non-Hispanic Black | 8.28 (1.88) | 14.74 (1.98) | 9.47 (0.83) |  |
| Non-Hispanic White | 21.33 (4.16) | 51.63 (4.33) | 71.60 (1.54) |  |
| Hispanic | 17.67 (2.39) | 9.77 (1.70) | 4.62 (0.45) |  |
| Other race | 52.71 (4.24) | 23.86 (2.53) | 14.31 (0.83) |  |
| Marital status, % (SE) |  |  |  | **<0.001** |
| Married | 51.88 (3.42) | 46.54 (2.28) | 55.96 (1.19) |  |
| Divorced | 6.50 (1.12) | 11.51 (1.52) | 10.49 (0.49) |  |
| Living alone | 41.62 (3.45) | 41.96 (2.05) | 33.55 (1.25) |  |
| Poverty income ratio, % (SE) | 1.19 (0.76,1.76) | 1.38 (0.83,2.60) | 3.51 (1.88,5.00) | **<0.001** |
| Energy (kcal) | 1849 (1355,2544) | 1966 (1477,2698) | 2068 (1543,2692) | **0.010** |
| Caffeine (mg) | 83 (8,186) | 99 (12,237) | 132 (33,252) | **<0.001** |
| Sleep trouble, % (SE) |  |  |  | 0.090 |
| Yes | 23.15 (2.26) | 28.13 (1.89) | 29.32 (0.85) |  |
| No | 76.85 (2.26) | 71.87 (1.89) | 70.68 (0.88) |  |
| MetS, % (SE) |  |  |  | **<0.001** |
| Yes | 34.14 (2.55) | 36.33 (2.11) | 29.35 (0.88) |  |
| No | 65.86 (2.55) | 63.67 (2.11) | 70.65 (0.88) |  |
| Elevated TG, % (SE) |  |  |  | 0.580 |
| Yes | 38.53 (2.53) | 37.32 (1.83) | 36.17 (0.96) |  |
| No | 61.47 (2.53) | 62.68 (1.83) | 63.83 (0.96) |  |
| Low HDL-C, % (SE) |  |  |  | **0.003** |
| Yes | 29.76 (2.49) | 33.91 (2.57) | 25.81 (0.85) |  |
| No | 70.24 (2.49) | 66.09 (2.57) | 74.19 (0.85) |  |
| Elevated FPG, % (SE) |  |  |  | **<0.001** |
| Yes | 37.00 (2.49) | 30.65 (2.06) | 24.71 (0.80) |  |
| No | 63.00 (2.49) | 30.65 (2.06) | 75.29 (0.80) |  |
| Elevated WC, % (SE) |  |  |  | 0.390 |
| Yes | 57.39 (2.74) | 57.97 (1.71) | 55.57 (1.11) |  |
| No | 42.61 (2.74) | 42.03 (1.71) | 44.43 (1.11) |  |
| Elevated BP, % (SE) |  |  |  | **<0.001** |
| Yes | 37.60 (2.82) | 38.31 (2.33) | 31.28 (0.84) |  |
| No | 62.40 (2.82) | 61.69 (2.33) | 68.72 (0.84) |  |
| OBS | 20 (15,26) | 19 (14,25) | 23 (17,28) | **<0.001** |
| Dietary OBS | 16 (10,21) | 15 (9-21) | 18 (13,23) | **<0.001** |
| Lifestyle OBS | 5 (4,5) | 4 (3,5) | 5 (4,6) | **<0.001** |

The P value was calculated by the Kruskal-Wallis H test for continuous variables. The P value was calculated by Chi-square test for categorial variables. SE, standard error; MetS, metabolic syndrome; TG, triglycerides; HDL-C, high-density lipoprotein cholesterol; FPG, fasting plasma glucose; WC, waist circumference; BP, blood pressure; OBS, oxidative balance score.

**Supplemental Table S7** Baseline characteristics of the participants stratified by poverty income ratio.

| Variable | <1 | 1-2 | 2-4 | >4 | P value |
| --- | --- | --- | --- | --- | --- |
| Age (years) | 38 (25,53) | 42 (29,58) | 47 (32,61) | 51 (38,61) | **<0.001** |
| Gender, % (SE) |  |  |  |  | **0.040** |
| Male | 47.36 (1.50) | 50.46 (1.27) | 51.78 (1.25) | 53.04 (1.10) |  |
| Female | 52.64 (1.50) | 49.54 (1.27) | 48.22 (1.25) | 46.96 (1.10) |  |
| Race/Ethnicity, % (SE) |  |  |  |  | **<0.001** |
| Non-Hispanic Black | 19.48 (2.16) | 13.09 (1.55) | 9.75 (1.07) | 5.28 (0.57) |  |
| Non-Hispanic White | 46.71 (3.46) | 58.83 (2.93) | 68.88 (2.13) | 80.72 (1.26) |  |
| Hispanic | 9.46 (1.31) | 7.45 (0.84) | 5.43 (0.61) | 2.96 (0.35) |  |
| Other race | 24.36 (2.00) | 20.93 (1.86) | 15.95 (1.30) | 11.05 (0.83) |  |
| Education levels, % (SE) |  |  |  |  | **<0.001** |
| Less than high school | 7.34 (0.85) | 6.05 (0.85) | 1.62 (0.33) | 0.11 (0.03) |  |
| High school diploma | 19.01 (1.74) | 12.34 (1.24) | 5.80 (0.63) | 2.11 (0.37) |  |
| More than high school | 73.65 (2.12) | 81.61 (1.66) | 92.58 (0.66) | 97.78 (0.37) |  |
| Marital status, % (SE) |  |  |  |  | **<0.001** |
| Married | 26.89 (2.00) | 41.16 (1.79) | 56.11 (1.56) | 70.06 (1.46) |  |
| Divorced | 13.02 (1.37) | 14.20 (1.17) | 11.53 (0.89) | 7.13 (0.63) |  |
| Living alone | 60.10 (2.58) | 44.64 (1.92) | 32.36 (1.42) | 22.81 (1.47) |  |
| Energy (kcal) | 2025 (1459,2758) | 1995 (1474,2643) | 2068 (1582,2702) | 2087 (1577,2675) | 0.340 |
| Caffeine (mg) | 79 (4,200) | 96 (15,210) | 135 (40,260) | 146 (50,264) | **<0.001** |
| Sleep trouble, % (SE) |  |  |  |  | 0.710 |
| Yes | 30.72 (1.98) | 27.94 (1.46) | 28.73 (1.62) | 29.34 (1.13) |  |
| No | 69.28 (1.98) | 72.06 (1.46) | 71.27 (1.62) | 70.66 (1.13) |  |
| MetS, % (SE) |  |  |  |  | 0.080 |
| Yes | 30.00 (1.97) | 32.09 (1.37) | 31.53 (1.37) | 27.88 (1.37) |  |
| No | 70.00 (1.97) | 67.91 (1.37) | 68.47 (1.37) | 72.12 (1.37) |  |
| Elevated TG, % (SE) |  |  |  |  | 0.690 |
| Yes | 36.10 (1.98) | 37.68 (1.26) | 36.43 (1.48) | 35.65 (1.09) |  |
| No | 63.90 (1.98) | 62.32 (1.26) | 63.57 (1.48) | 64.35 (1.09) |  |
| Low HDL-C, % (SE) |  |  |  |  | **<0.001** |
| Yes | 31.85 (1.88) | 33.43 (1.57) | 26.77 (1.27) | 21.37 (1.09) |  |
| No | 68.15 (1.88) | 66.57 (1.57) | 73.23 (1.27) | 78.63 (1.09) |  |
| Elevated FPG, % (SE) |  |  |  |  | 0.490 |
| Yes | 23.72 (1.42) | 25.25 (1.20) | 26.95 (1.60) | 25.02 (1.34) |  |
| No | 76.28 (1.42) | 74.75 (1.20） | 73.05 (1.60) | 74.98 (1.34) |  |
| Elevated WC, % (SE) |  |  |  |  | 0.100 |
| Yes | 54.87 (2.32) | 58.74 (1.37) | 57.27 (1.36) | 53.66 (1.78) |  |
| No | 45.13 (2.32) | 41.26 (1.37) | 42.73 (1.36) | 46.34 (1.78) |  |
| Elevated BP, % (SE) |  |  |  |  | **0.010** |
| Yes | 26.18 (1.89) | 32.26 (1.42) | 32.07 (1.39) | 33.56 (1.09) |  |
| No | 73.82 (1.89) | 67,74 (1.42) | 67.93 (1.39) | 66.44 (1.09) |  |
| OBS | 20 (14,26) | 21 (15,27) | 22 (17,27) | 24 (19,29) | **<0.001** |
| Dietary OBS | 16 (10,21) | 17 (11,22) | 18 (13,22) | 19 (14,24) | **<0.001** |
| Lifestyle OBS | 4 (3,5) | 4 (3,5) | 5 (4,6) | 5 (4,6) | **<0.001** |

The P value was calculated by the Kruskal-Wallis H test for continuous variables. The P value was calculated by Chi-square test for categorial variables. SE, standard error; MetS, metabolic syndrome; TG, triglycerides; HDL-C, high-density lipoprotein cholesterol; FPG, fasting plasma glucose; WC, waist circumference; BP, blood pressure; OBS, oxidative balance score.

**Supplemental Table S8** Baseline characteristics of the participants stratified by poverty income ratio.

| Variable | Sleep trouble | No sleep trouble | P value |
| --- | --- | --- | --- |
| Age (years) | 52 (38,63) | 44 (31,58) | **<0.001** |
| Gender, % (SE) |  |  | **<0.001** |
| Male | 44.84 (1.12) | 54.20 (0.79) |  |
| Female | 55.16 (1.12) | 45.80 (0.79) |  |
| Race/Ethnicity, % (SE) |  |  | **<0.001** |
| Non-Hispanic Black | 8.92 (0.91) | 10.19 (0.96) |  |
| Non-Hispanic White | 75.44 (1.77) | 66.17 (1.82) |  |
| Hispanic | 4.12 (0.57) | 5.83 (0.53) |  |
| Other race | 11.53 (1.05) | 17.81 (1.08) |  |
| Education levels, % (SE) |  |  | 0.090 |
| Less than high school | 2.05 (0.25) | 2.79 (0.29) |  |
| High school diploma | 6.99 (0.73) | 7.32 (0.57) |  |
| More than high school | 90.96 (0.81) | 89.89 (0.73) |  |
| Marital status, % (SE) |  |  | **0.003** |
| Married | 51.97 (1.74) | 56.49 (1.26) |  |
| Divorced | 12.88 (0.95) | 9.46 (0.52) |  |
| Living alone | 35.15 (1.58) | 34.04 (1.31) |  |
| Poverty income ratio, % (SE) | 3.22 (1.57,2.00) | 3.29 (1.65,5.00) | 0.970 |
| Energy (kcal) | 1984 (1489,2604) | 2080 (1553,2714) | **0.010** |
| Caffeine (mg) | 144 (45,271) | 119 (29,243) | **<0.001** |
| MetS, % (SE) |  |  | **<0.001** |
| Yes | 38.34 (1.14) | 26.55 (1.02) |  |
| No | 61.66 (1.14) | 73.45 (1.02) |  |
| Elevated TG, % (SE) |  |  | **<0.001** |
| Yes | 40.52 (1.20) | 34.58 (1.04) |  |
| No | 59.48 (1.20) | 65.42 (1.04) |  |
| Low HDL-C, % (SE) |  |  | 0.080 |
| Yes | 28.39 (1.40) | 25.72 (0.84) |  |
| No | 71.61 (1.40) | 74.28 (0.84) |  |
| Elevated FPG, % (SE) |  |  | **<0.001** |
| Yes | 30.75 (1.05) | 23.28 (0.90) |  |
| No | 69.25 (1.05) | 76.72 (0.90) |  |
| Elevated WC, % (SE) |  |  | **<0.001** |
| Yes | 63.51 (1.41) | 52.63 (1.11) |  |
| No | 36.49 (1.41) | 47.37 (1.11) |  |
| Elevated BP, % (SE) |  |  | **<0.001** |
| Yes | 42.27 (1.42) | 27.72 (0.92) |  |
| No | 57.73 (1.42) | 72.28 (0.92) |  |
| OBS | 22 (17,27) | 23 (17,28) | 0.060 |
| Dietary OBS | 18 (12,23) | 18 (13,23) | 0.590 |
| Lifestyle OBS | 5 (3,5) | 5 (4,6) | **<0.001** |

The P value was calculated by the Kruskal-Wallis H test for continuous variables. The P value was calculated by Chi-square test for categorial variables. SE, standard error; MetS, metabolic syndrome; TG, triglycerides; HDL-C, high-density lipoprotein cholesterol; FPG, fasting plasma glucose; WC, waist circumference; BP, blood pressure; OBS, oxidative balance score.


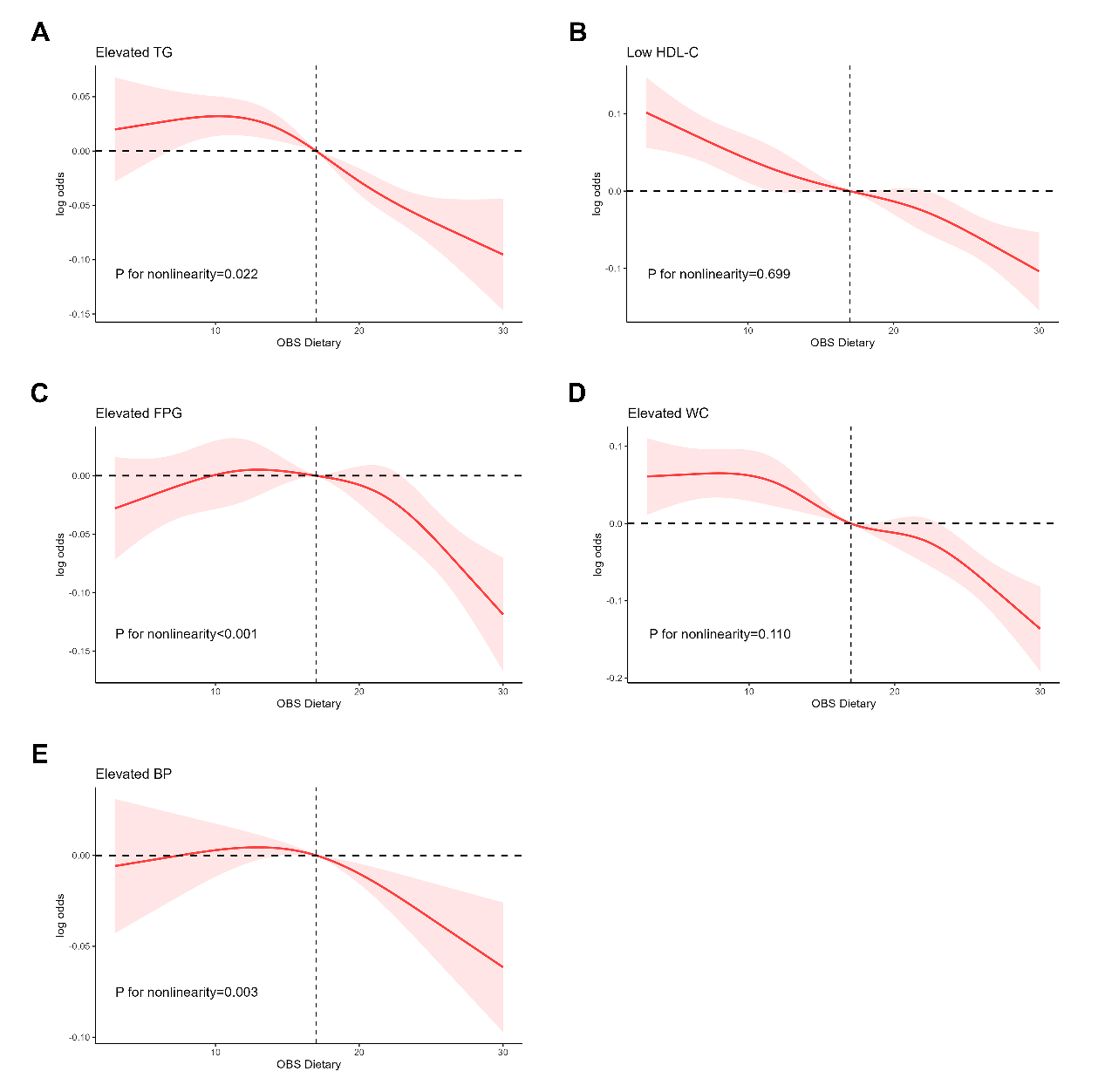


**Supplemental Figure 2.** Nonlinear relationship of dietary OBS with five components of MetS. **(A)** Nonlinear relationship between dietary OBS and elevated TG. **(B)** Nonlinear relationship between dietary OBS and low HDL-C. **(C)** Nonlinear relationship between dietary OBS and elevated FPG. **(D)** Nonlinear relationship between dietary OBS and elevated WC. **(E)** Nonlinear relationship between dietary OBS and elevated BP. OBS, oxidative balance score; MetS, metabolic syndrome; TG, triglyceride; HDL-C, high density cholesterols; WC, waist circumference; BP, blood pressure.


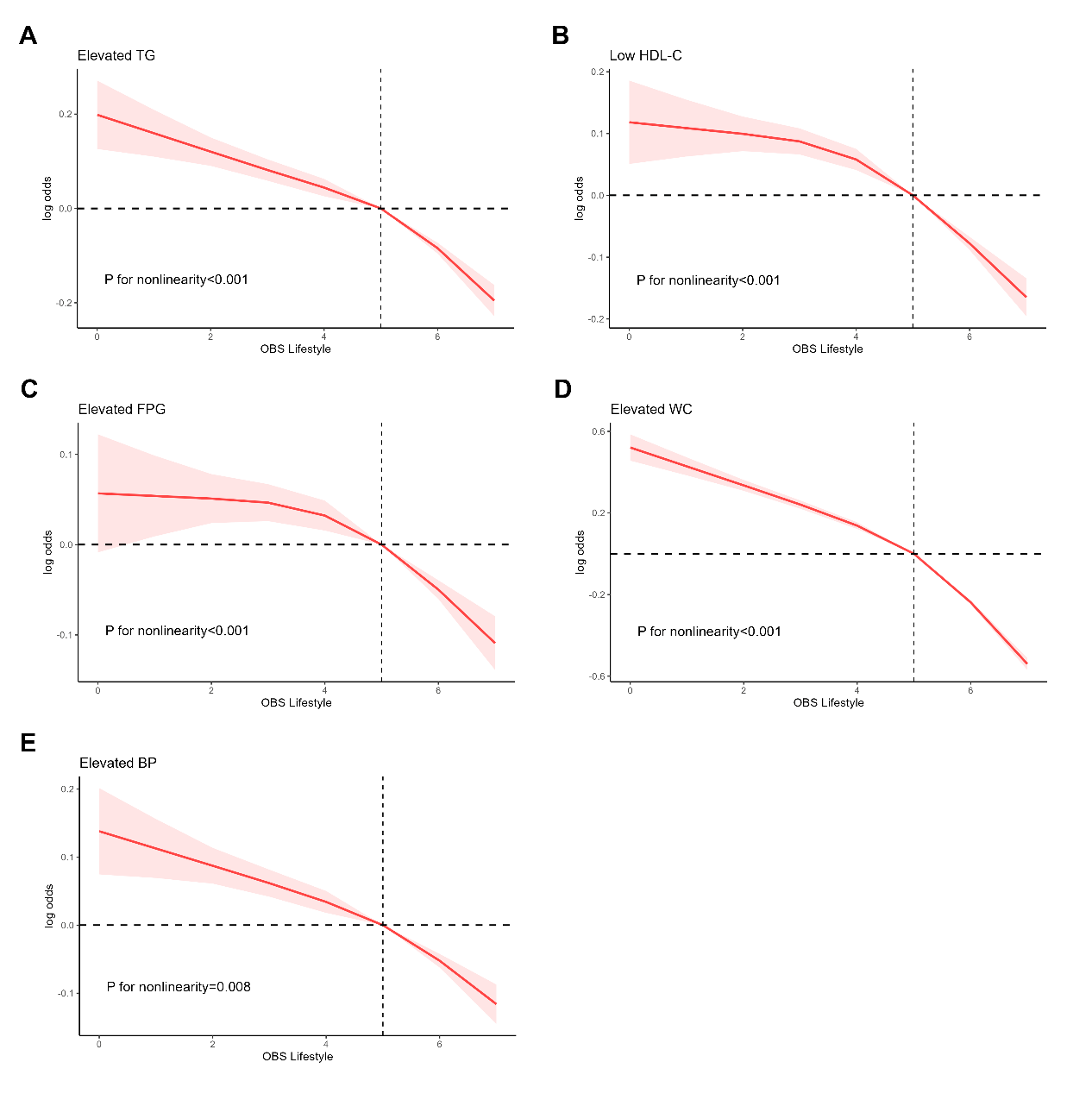


**Supplemental Figure 3.** Nonlinear relationship of lifestyle OBS with five components of MetS. **(A)** Nonlinear relationship between lifestyle OBS and elevated TG. **(B)** Nonlinear relationship between lifestyle OBS and low HDL-C. **(C)** Nonlinear relationship between lifestyle OBS and elevated FPG. **(D)** Nonlinear relationship between lifestyle OBS and elevated WC. **(E)** Nonlinear relationship between lifestyle OBS and elevated BP. OBS, oxidative balance score; MetS, metabolic syndrome; TG, triglyceride; HDL-C, high density cholesterols; WC, waist circumference; BP, blood pressure.
